# Intrapartum fetal deaths and unexpected neonatal deaths in the Republic of Ireland: 2011 – 2014; a descriptive study

**DOI:** 10.1186/s12884-017-1636-6

**Published:** 2018-01-04

**Authors:** K. McNamara, K. O’Donoghue, R. A. Greene

**Affiliations:** 1 Pregnancy Loss Research Group, Department of Obstetrics and Gynaecology, University College Cork, Cork, Ireland; 2 The Irish Centre for Fetal and Neonatal Translational Research (INFANT), University College Cork, Cork, Ireland; 3 The National Perinatal Epidemiology Centre, University College Cork, Cork, Ireland; 4 Department of Obstetrics and Gynaecology, Cork University Maternity Hospital, 5th Floor, Wilton, Cork, Ireland

**Keywords:** Intrapartum fetal death, Cause of death, Confidential enquiry

## Abstract

**Background:**

Intrapartum fetal death, the death of a fetus during labour, is a tragic outcome of pregnancy. The intrapartum death rate of a country is reflective of the care received by mothers and babies in labour and it is through analysing these cases that good aspects of care, as well as areas for improvement can be identified. Investigating unexpected neonatal deaths that may be associated with an intrapartum event is also helpful to fully appraise intrapartum care. This is a descriptive study of intrapartum fetal deaths and unexpected neonatal deaths in Ireland from 2011 to 2014.

**Methods:**

Anonymised data pertaining to all intrapartum fetal deaths and unexpected neonatal deaths for the study time period was obtained from the national perinatal epidemiology centre. All statistical analyses were conducted using Statistical package for the Social Sciences (SPSS).

**Results:**

There were 81 intrapartum fetal deaths from 2011 to 2014, and 36 unexpected neonatal deaths from 2012 to 2014. The overall intrapartum death rate was 0.29 per 1000 births and the corrected intrapartum fetal death rate was 0.16 per 1000 births. The overall unexpected neonatal death rate was 0.17 per 1000 live births. Major Congenital Malformation accounted for 36/81 intrapartum deaths, chorioamnionitis for 18/81, and placental abruption accounted for eight babies’ deaths. Intrapartum asphyxia accounted for eight of the intrapartum deaths. With respect to the neonatal deaths over half (21/36, 58.3%) of the babies died as a result of hypoxic ischaemic encephalopathy. Information is also reported on both maternal and individual baby demographics.

**Conclusions:**

This is the first detailed descriptive analysis of intrapartum deaths and unexpected intrapartum event related neonatal deaths in Ireland. The corrected intrapartum fetal death rate was 0.16 per 1000 births. Despite our results being based on the best available national data on intrapartum deaths and unexpected neonatal deaths, we were unable to identify if any of these deaths could have been prevented. A more formal confidential inquiry based system is necessary to fully appraise these cases.

## Background

Intrapartum fetal death, the death of a fetus during labour is a tragic and traumatic outcome of pregnancy [1]. Globally intrapartum fetal deaths exert a massive healthcare burden and it is estimated that approximately 1.3 million infants die each year during labour [2]. The number of intrapartum fetal deaths (IPDs) that occur in high income countries is small (0.3 – 0.7/1000 births) [2, 3] but each one leaves a profound impact, not just on the parents but also on the healthcare professionals involved [4, 5].

It is widely accepted that the intrapartum death rate of a particular hospital or country is reflective of the care received by mothers and infants in labour and that access to and utilisation of high quality, evidence-based intrapartum care is one way to further reduce intrapartum death rates [2, 3, 6–10]. It is only through analysing these cases that good aspects of care, as well as areas for improvement can be identified [11]. In addition, investigating unexpected neonatal deaths that may be associated with an intrapartum event is helpful to fully appraise intrapartum care, and evidence shows that improved intrapartum care can also reduce unexpected neonatal deaths [12, 13].

For these reasons we decided to collect and analyse data pertaining to all IPDs (normally formed and anomalous) and unexpected neonatal deaths of infants born after 34 weeks of gestational age that occurred in the ROI between the years 2011 and 2014. The aims of this study were; to identify the IPD rate during the time period studied; to describe both maternal, fetal and neonatal demographics pertaining to antenatal, intrapartum and postpartum care; to ascertain causation and to identify if any or all or none of these cases could have been prevented.

In 2010, the National Perinatal Epidemiology Centre (NPEC) began to collect, analyse and audit data pertaining to all births in the ROI [10], and as such this is the first time a study like this has been attempted in this country. A previous study conducted by Walsh et al. [9] identified trends in intrapartum deaths in three large Dublin maternity hospitals over a 20-year period, but ours is the first descriptive study from Ireland that uses national perinatal data, obtained from NPEC to accurately describe the national intrapartum death rate, underlying maternal and fetal/neonatal demographics, available intrapartum details, postnatal investigations and causes of death.

## Methods

### Design and setting

Since 2010 the National Perinatal Epidemiology Centre (NPEC) has been collecting and auditing anonymised data on all stillbirths and neonatal deaths that occur in the original 20, but since 2014, 19 maternity units in the Republic of Ireland [10]. A stillbirth in Ireland is defined as a baby delivered without signs of life from 24 weeks gestation or with a birth weight equal to or greater than 500 g, while an early neonatal death is the death of a live born baby within 7 completed days of birth [10]. Data is collected by nominated individuals, either obstetricians or midwives directly from the patient charts in each maternity unit and sent to NPEC via their online Perinatal Mortality Notification Form (www.ucc.ie/en/media/research/nationalperinatalepidemiologycentre/forms/PerinatalMortalityForm2017.pdf). This is a standardised notification form that is based on the previously validated Centre for Maternal and Child Enquiries (CMACE) Perinatal Death Notification Form [14]. Using this form, the nominated individuals in each hospital are asked to identify any maternal, fetal or neonatal conditions or complications that may have contributed to the infant’s death. These individuals are also requested to document the main cause of death while referencing the post-mortem and placental pathology reports, if available. These anonymised data are stored electronically in the NPEC database and form the basis of the clinical perinatal mortality reports produced by NPEC each year [10].

Since 2014, the NPEC have been consolidating their data with that of the National Perinatal Reporting System (NPRS) which is the perinatal surveillance body in Ireland concerned with the collection and reporting of all births in the Republic of Ireland. From 2011 to 2014 all stillbirths and neonatal deaths were cross-referenced off published annual hospital reports. It is possible, therefore that some cases may be unaccounted for, but as the NPEC data represents the most complete dataset with respect perinatal deaths this is why it was used.

For the purposes of this study, a request was made to the NPEC data access committee, to grant access to the information stored on all of the intrapartum deaths and unexpected neonatal deaths that occurred in the ROI between 2011 and 2014. These years were chosen as the database for this time period was likely to contain the most complete data. We defined intrapartum fetal death as the death of any infant occurring in labour, while an unexpected neonatal death referred to any infant that died in the early neonatal period, born at a gestational age of more than 34 weeks, or with a birth weight of more than 2500 g (that was not secondary to a known major congenital malformation). These cut offs were chosen by the national perinatal epidemiology centre, as infants born after this gestational age and above this weight cut off are expected to survive.. Data on intrapartum deaths were available for the four years in question but for the unexpected neonatal deaths data were available for 2012-2014 only.

### Statistical analysis

All statistical analyses were conducted using Statistical package for the Social Sciences (SPSS) version 22. This is a descriptive study and all maternal and infant characteristics are presented in detail. Where appropriate for continuous data variables, and where the data is normally distributed, the mean and standard deviation are reported. Where the data is not normally distributed the median and interquartile range (IQR) are presented. In order for our intrapartum death rates to be compared off international data we have presented two figures. Firstly an uncorrected rate which represents all intrapartum deaths in Ireland and secondly a corrected rate which is calculated after all infants who died secondary to a major congenital malformation were removed.

## Results

There were 81 intrapartum fetal deaths from 2011 to 2014, and 36 unexpected neonatal deaths from 2012 to 2014.. The overall IPD rate was 0.29 per 1000 births. When this rate was corrected for infants with a major congenital malformation it was 0.16 per 1000 total births. The overall unexpected neonatal death rate was 0.17 per 1000 live births. The individual death rates for each year are presented in Table 1.

**Table 1:** Individual IPD Rates and Unexpected Neonatal Death (uNND) Rates

	2011	2012	2013	2014	Total
Total Births	74,265	71,755	69,146	67,663	282,829
IPDs^c^	24	15	23	19	81
uNND^b^	–	11	11	14	36^d^
IPD rate/ 1000 total births (corrected^a^)	0.32 (0.19)	0.20 (0.12)	0.33 (0.21)	0.28 (0.11)	0.29 (0.16)
uNND rate/ 1000 live births	–	0.15	0.16	0.20	–

^a^Corrected for major congenital malformation. ^b^*uNND* unexplained neonatal death, ^c^*IPD* intrapartum deaths, ^d^total uNNDs excluding 2011

### Maternal characteristics

During our study period, and based on our inclusion criteria, 117 mothers delivered an infant who died during labour or in the early neonatal period. The mean maternal age was 31 years. Ethnicity was reported in 115 of the 117 mothers in this cohort. The majority (*n* = 97, 82.9%) were of white Irish origin. Occupation was documented for 105 of the 117 mothers, with 10% (*n* = 12) being unemployed at the time of their pregnancy. At their booking visit 25 (21.4%) mothers smoked, with 16 continuing to do so for the duration of the pregnancy. Smoking status at booking was not recorded for 12 (10%) of the mothers. The median BMI was 25 kg/m^2^, with an IQR of 7.2 kg/m^2^.. In total, 42% (*n* = 49) of mothers in this cohort were overweight or obese. Additional data on the maternal age range, ethnicity and BMI are presented in Table 2.

**Table 2:** Maternal Demographics

	IPDs (*N* = 81, %)	Neonatal Deaths (*N* = 36, %)	All Deaths (*N* = 117, %)
Age Group (years)			
< 20	3 (3.7)	3 (8.3)	6 (5.1)
20 – 24	15 (18.5)	2 (5.6)	17 (14.5)
25 – 29	11 (13.6)	5 (13.9)	16 (13.7)
30 – 34	25 (30.9)	16 (44.4)	41 (35)
35 – 39	22 (27.2)	6 (16.7)	28 23.9)
> 40	5 (6.2)	4 (11.1)	9 (7.7)
Total	81 (100)	36 (100)	117 (100)
Ethnic Group			
White Irish	66 (81.5)	31 (86.1)	97 (82.9)
Irish traveller	2 (2.5)	0	2 (1.7)
Other white	7 (8.6)	3 (8.3)	10 (8.5)
Asian	2 (2.5)	1 (2.8)	3 (2.6)
Black	2 (2.5)	0	2 (1.7)
Other Mixed	0	1 (2.8)	1 (0.9)
Not recorded/missing	2 (2.5)	0	2 (1.7)
Total	81 (100)	36 (100)	117 (100)
BMI^a^ (kg/m^2^)			
Underweight (<18.5)	1 (1.2)	0	1 (0.8)
Healthy (18.6 – 24.9)	37 (45.7)	10 (27.8)	47 (40.2)
Overweight (25- 29.9)	20 (24.7)	9 (25)	29 (24.8)
Obese (>30)	9 (11.1)	11 (30.6)	20 (17.1)
Not recorded/missing	14 (17.3)	6 (16.7)	20 (17.1)
Total	81 (100)	36 (100)	117 (100)

^a^*BMI* Body Mass Index

A third (39/117, 33.3%) of mothers had a documented pre-existing medical condition. Some mothers had more than one medical condition while no information about past medical history was given on others. The most common medical conditions were: diabetes (4/39, 10.2%), other endocrine disorders (3/39, 7.7%) psychiatric conditions (2/39, 5.1%), and epilepsy (2/39, 5.1%). With respect to their past obstetric history, for 41 mothers this was their first pregnancy. Of the remaining 76 mothers, 35 (29.9%) had at least one prior miscarriage with three having had three or more miscarriages. There were 11 mothers in our cohort who had at least one previous Caesarean section. In terms of previous pregnancy complications, a minority of mothers (*n* = 3, 2.5%)) had other infants with congenital abnormalities, one mother had a past history of pre-eclampsia while another mother had a previous stillbirth.

Gestational Age at booking in pregnancy was unknown or was missing from the dataset for 22 (18.8%) of the mothers. Almost 60% (69) booked at a gestational age of less than 16 weeks with the remaining 23% (26) booking after 16 weeks of gestational age.

Labour commenced spontaneously in 68% (80/117) of mothers, 23% (27/117) had their labours induced while the remaining 8.5% (10) underwent a pre-labour emergency Caesarean Section (CS).

The presentation at delivery was recorded for 111 of the 117 infants. Most infants were in a vertex presentation at delivery (77, 65.8%), while 32 (27.4%) presented breech at delivery.

With respect to the mode of delivery, six mothers had a pre-existing plan for delivery by CS. Of these two had assisted breech deliveries at very preterm gestations and the remaining four had a CS after the onset of labour, one of which was following an unsuccessful instrumental delivery. Additional data on the mode of delivery are presented in Table 3.

**Table 3:** Mode of Delivery

Mode of delivery	IPDs (*n* = 81, %)	NNDs (*n* = 36, %)	All deaths (*n* = 117, %)
SVD	36 (44.4)	8 (22.2)	44 (37.9)
Vacuum	3 (3.7)	6 (16.7)	9 (7.7)
Forceps	4 (4.9)	0	4 (3.5)
AB delivery	28 (34.6)	0	28 (23.9)
CS pre-labour	2 (2.5)	8 (22.2)	10 (8.5)
CS after onset of labour	8 (9.9)	14 (38.9)	22 (18.8)
Total	81 (100)	36 (100)	117 (100)

*SVD* Spontaneous vaginal delivery, *AB delivery* Assisted Breech Delivery, *CS* Caesarean section

### Fetal and neonatal characteristics

There were more male infants than female infants in this cohort (Male 62, 53%, Female 54, 46.2%). One intrapartum death was associated with an infant of indeterminate sex. The majority of pregnancies were singleton pregnancies. In total four of the infants born were of a twin pair, two from a dichorionic diamniotic twin pregnancy, one from a monochorionic diamniotic twin pregnancy and one from a twin pregnancy of unknown chorionicity. The outcomes of the surviving twins are not known.

With respect to gestational age the majority of intrapartum fetal deaths (57/81, 70.4%) occurred in infants at opposite ends of the gestational age spectrum: twenty-eight deaths occurred between 22 and 27^+6^ weeks of gestational age, while a further twenty-nine deaths occurred after 37 weeks of gestational age. When infants with a major congenital malformation were excluded (35/81), the predominant gestational age range of the infants who died during labour was 22 to 27^+6^ weeks (26, 56.5%).

**Fig. 1:**
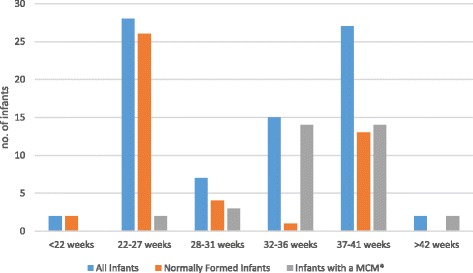
Gestational age ranges of infants (*N* = 117). *Major Congenital Malformation

Gestational age was unknown for three of the infants who died in the neonatal period. The remaining 33 infants were delivered after 34 weeks of gestational age, with 27/36 (75%) being born after 37 weeks (Fig. 1).

The median birth weight for all infants was 2280 g with an IQR of 2424 g. The median birth weight of all infants who had an IPD was 1300 g with an IQR of 1745 g while the mean birth weight of all infants who died in the neonatal period was 3370 g with a standard deviation of 579 g.

Of the 82 normally formed infants 20.7% (17/82) had a customised birth weight less than the 10th percentile for gestational age. The Gestation Related Optimum Weight (GROW) software was used to calculate these centiles [15]. Complete data was missing for three of the infants and their birth weight centiles could not be calculated. Growth restriction was suspected antenatally for only one of the infants. Most infants (28/35, 80%) with a major congenital malformation had a birth weight less than the 10th percentile for gestation. Overall, 40/117 infants were normally formed and had a gestational age of more than 37 weeks. When GROW centiles were calculated 7 of the 40 (17.5%) normally formed infants had a birth weight less than the 10th percentile for gestation. This was suspected antenatally for four of the infants.

### Postnatal investigations

In our cohort of infants the post-mortem rate was 47.9% (56/117) with a further 43.6% (51/117) of parents being offered the choice to proceed with a post-mortem examination on the infant and declining. It is not clear from the dataset why post-mortems were declined for this group of infants, or whether the Coroner was contacted in any case. By law in Ireland, all unnatural stillbirths and intrauterine deaths must be reported to the local coroner. It is usual practice, therefore, that all normally formed intrapartum deaths are reported to the coroner.

The post-mortem rate for infants who died in the neonatal period was higher (34/36, 94.4%) than for those who died during labour (27.2%). Of the normally formed infants, 61% (50/82) underwent a post-mortem examination while 92% (37/40) of infants who were normally formed and had a gestational age of more than 37 weeks had a post-mortem. It cannot be interpreted from the data how many cases were referred to the Coroner. Table 4 describes the post-mortem examination rates and placental histology rates for each group of infants.

**Table 4:** Post-Mortem Rates

Investigation	IPDs (*n* = 81, %)	NNDs (*n* = 36, %)	All deaths (*n* = 117, %)
Coroner’s Case	9 (11.1)	31 (86.1)	40 (34.2)
Total PM	22 (27.2)	34 (94.4)	56 (47.9)
Offered and declined	49 (60.5)	2 (5.6)	51 (43.6)
Placental Histology	76 (93.8)	31 (86.1)	107 (91.5)
Investigation	Infants, Major congenital malformation (*n* = 35, %)	Normally formed (*n* = 82, %)	Normally formed >37/40 (*n* = 40)
Coroner’s Case	0	40 (48.4)	33 (82.5)
Total PM	6 (17.1)	50 (61)	37 (92.5)
Offered and declined	22 (62.9)	29 (35.4)	3 (7.5)
Placental Histology	30 (85.7)	77 (93.9)	36 (90)

*PM* Post-mortem examination

Placental histology was available in 91% (107/117) of cases. Specific placental pathology was identified in over 50% of cases. Some placentas had more than one documented pathology and the different pathologies are documented in Table 5. There was no universal structure to the way placental pathology reports were documented. Some collaborators entered the full pathology report as a free text to allow the reviewers in NPEC to decipher the report while others completed the form with minimal data. It is not clear from the dataset if this was because the original report contained minimal data or not.

**Table 5:** Placental Pathology^a^

Investigation	IPDs (*n* = 81, %)	NNDs (*n* = 36, %)	All deaths (*n* = 117, %)
Velamentous Insertion	2 (2.5)	2 (5.6)	4 (3.4)
Vasa Praevia	0	3 (8.3)	3 (2.6)
Placental Infarction	1 (1.2)	0	1 (0.9)
Chorioamnionitis	17 (21)	3 (8.3)	20 (17.1)
Fetal Vasculitis	2 (2.4)	2 (4.2)	4 (3.4)
Retroplacental haemorrhage	8 (9.9)	1 (2.8)	9 (7.7)
Villitis	1 (1.2)	3 (8.3)	4 (3.4)
Other	19 (23.5)	13 (36.1)	32 (27.4)
No findings	35 (43.2)	10 (27.8)	45 (38.5)

^a^does not equall 100% as some placentas had multiple documented pathologies

Of the deaths that occurred in normally formed infants (82/117), 52 underwent local hospital review (63.4%) and of those with a major congenital malformation (35/117) 15 had a hospital review (42.9%). Of those infants who were normally formed and delivered after 37 weeks gestational age (40/117) 33 had a local hospital review (82.5%). From the dataset it was unclear as to why these reviews did not take place for all cases.

### Cause of death – Intrapartum fetal deaths (*N* = 81)

Table 6 lists the main causes of death for all infants who died during labour. In total, 36/81 infants were diagnosed with a major congenital malformation, but one of these infants (Trisomy 21) died as a result of severe chorioamnionitis. Of the remaining 35, abnormalities in the central nervous system (16/35, 45.7%) and chromosomal abnormalities (15/35, 42.9%) were given as the most common reasons why the infant died.

**Table 6:** Main Cause of Death

Cause of Death	(*n*, %)
Major congenital malformation	35 (43.2)
Chorioamnionitis	18 (22.2)
APH from a placental Abruption	8 (9.9)
Intrapartum Asphyxia	8 (9.9)
Unexplained	4 (5)
Specific placental	3 (3.7)
Mechanical	2 (2.5)
APH from a placenta praevia	1 (1.2)
Cord accident	1(1.2)
Associated Obstetric factors (PPROM)	1 (1.2)
Total	81 (100)

*APH* Antepartum haemorrhage, *PPROM* Preterm Prelabour rupture of membranes

Chorioamnionitis was reported as the main cause of death in 18 of the remaining infants. With the exception of two infants, all were born at a gestational age of less than 28 weeks. The first of the term infants died at 41 weeks of gestational age following a ventouse delivery. This was the infant that at postmortem was found to have trisomy 21. The second of these infants died following an induction of labour and ventouse delivery at 41 + 5 weeks gestational age. A hospital post-mortem examination was performed, and the cause of death was reported as severe chorioamnionitis and meconium aspiration with ensuing asphyxia. It was impossible to ascertain from the dataset if chorioamnionitis was suspected during these mothers’ labours or not.

Antepartum haemorrhage from a placental abruption was another common cause of death, accounting for eight infants’ deaths. Only one infant died at term; the others were all less than 28 weeks of gestational age. The term infant was delivered by forceps after an induced labour at 37 + 6 weeks of gestation. A post-morterm examination was not undertaken but placental histology agreed with the clinical diagnosis of placental abruption.

Placental lesions were identified in 50/81, (56.8%) of the infants who died but were only classed as the main cause of death in three cases. The specific pathologies included maternal vascular malperfusion and fetal vasculitis. One of these infants was born at 40^+8^ weeks of gestational age, the others were both born prior to 30 weeks.

Intrapartum asphyxia accounted for eight of the intrapartum deaths. Coroner’s Post-mortems were carried out in five of the cases, a hospital post-mortem in one case and in the remaining two cases parents were offered post- mortem examinations but declined. The majority, (6/8) had some other contributing condition: Uterine rupture, premature prelabour rupture of the membranes (two cases), cord accident, placental lesion, and fetal growth restriction.

Of the remaining eight infants, one died secondary to antepartum haemorrhage from placenta praevia, one died following a preterm prelabour rupture of membranes (PPROM), two died as a result of mechanical causes (uterine anomalies and cord prolapse) and four deaths were unexplained. Post-mortem examinations were conducted in three of the four unexplained cases.

### Cause of death - Intrapartum event related neonatal deaths (*N* = 36)

Over half (21/36, 58.3%) of the infants died as a result of hypoxic ischaemic encephalopathy (HIE). The majority of these infants were delivered at a gestational age of greater than 37 weeks (19/21). With respect to labour, 12/21 were spontaneous labours and 4/21 were induced labours. The most common mode of delivery was Caesarean Section (15/21). Five of the CS were pre-labour while the rest were conducted after the onset of labour*.* There was one unsuccessful instrumental delivery that was then converted to a CS in this group.

The mean birth weight at delivery was 3526 g with a standard deviation of 598 g*.* Utilising the GROW software to predict centiles, 3 infants were small for gestational age (<10th percentile). It is not clear if this was suspected antenatally or not.

Most (19/21) infants in this group had a post-mortem examination with 18 being directed by the Coroner.

The main associated obstetric issues are detailed below in Table 7.

**Table 7:** Characteristics of Infants who Died Secondary to HIE

Gestational Age	Birth weight centile	Mode of Delivery	Obstetric factors
34 + 4	50th – 89th	SOL and EMCS	Placental-MVM, spontaneous PTL
35 + 6	>90th	SOL and EMCS	Placental-DVI, hx mat smoking, endocrine and inflammatory disease
36 + 1	50th – 89th	Pre-labour CS	APH- vasa praevia
36 + 3	10th – 49th	Pre-labour CS	Abruption
38 + 4	<10th	Pre-labour CS	Other maternal disorder- haemoperitoneum
39 + 0	50th – 89th	Perimortem CS	Maternal cardiac arrest at home, maternal, maternal death
39 + 2	50th – 89th	SOL and EMCS	Unexplained
39 + 6	50th – 89th	IOL and CS after onset of labour,	APH -Vasa praevia
40 + 0	50th – 89th	IOL and SVD	Placental- fetal thrombotic vasculopathy, abnormal uncoiled cord
40 + 0	50th – 89th	SOL and SVD	Nuchal cord, FBS result <7.25 intrapartum
40 + 1	10th – 49th	SOL and Ventouse	Uterine Rupture
40 + 3	10th – 49th	SOL and EmCS	Uterine rupture-previous vaginal deliveries only
40 + 3	<10th (<3rd)	SOL and EM CS	Pathological CTG, meconium, placenta- severe chorio and fetal vasculitis, PM mec aspiration
40 + 5	10th – 49th	Pre-labour CS	Placental – DVI/ umbilical cord haematoma
40 + 5	50th – 89th	SOL and vacuum delivery	Placental – DVI, mild choriodeciduitis
40 + 6	<3rd	Pre-labour CS	Placental – DVI
41 + 2	10th – 49th	Pre-labour CS	Hypercoiled cord
41 + 2	10th – 49th	SOL and EMCS	APH -Vasa praevia
41 + 2	50th – 89th	SOL and EMCS	Unexplained
41 + 3	>90th	IOL and SVD	Shoulder dystocia (IOL post dates)
41 + 5	50th – 89th	IOL, unsuccessful instrumental and EMCS	Unexplained

There were six unexplained neonatal deaths between 2012 and 2014. Further, at the time of entry into the NPEC database four cases were still waiting a post-mortem report from the Coroner.

In total there were nine other neonatal deaths secondary to intrapartum events. Perinatal infection was responsible for six of these. Two infants were delivered at home to mothers who were unbooked to any maternity unit; the gestational ageof both infants was unknown. The first weighed 2500 g at delivery; there was a suspicion of prolonged rupture of membranes and the placenta revealed acute chorioamnionitis. The second infant weighed 3070 g and Coronial post-mortem report concluded the cause of death was secondary to Group B Streptococcal septic shock.

Another infant was born to a mother who underwent a pre-labour CS at 34 + 1 weeks of gestational age. It is unclear why this mother had a CS at this gestation. A hospital post-mortem revealed congenital toxoplasmosis and placental histology confirmed this diagnosis.

Another infant died following a ventouse delivery at 38 + 5 weeks of gestational age and a coroner directed post-mortem PM confirmed Escerichia. coli sepsis. Another infant died from a gram-negative meningitis. This infant was delivered by CS, for a breech presentation at 39 + 2 weeks gestation following spontaneous onset of labour. A sixth infant died from acute chorioamnionitis following a ventouse delivery.

The three remaining infants died from a range of other conditions. One following a ruptured vasa praevia. The second of these has been attributed to Sudden Infant Death Syndrome (SIDS) and had no identifiable antecedant or obstetric factors. This was an infant that was well at birth following spontaneous vaginal delivery but died at 10 h of age. The last infant died at day zero of life, following a ventouse delivery at 41 + 6 weeks of gestational age. This baby was small for gestational age, less than the 10th percentile for birth-weight. The coroner directed post-mortem for this infant revealed extensive traumatic intracranial haemorrhage, bilateral parietal and parietotemporal fractures, and soft cranial bones with prominent cranio-lacunae and ostepaenia. “Fracture” was the coded cause of death.

## Discussion

This is the first detailed descriptive analysis of intrapartum deaths and unexpected intrapartum event related neonatal deaths in the Republic of Ireland.

The corrected intrapartum fetal death rate of 0.16 per 1000 births in this study compares favourably with that of the United Kingdom (0.35) [16] and other high-income countries [2] but given the differences in maternal demographics that exist internationally, as well as the differing definitions of stillbirth, and hence intrapartum stillbirth, it is difficult to draw any conclusion from this figure alone [2]. It has, however, been recognised that in countries where women receive good quality intrapartum care that the proportion of intrapartum deaths is less than 10% of all stillbirths [6]. Since 2011 there have been 1253 stillbirths in the ROI (4.4 per 1000 live births, uncorrected for major congenital malformations) and intrapartum deaths make up 6.4% of all cases. While these figures point towards good overall maternity care, this study has revealed interesting aspects of both maternal and infant demographics and it has identified areas for improvement in antenatal care and post-mortem investigations.

Maternal smoking, obesity and timely booking to a hospital or a midwife in the pregnancy are all areas that need to be improved upon. In this study 21% of mothers smoked, while 42% were either over-weight or obese. Over one fifth of the mothers who experienced an intrapartum fetal death or unexpected neonatal death booked late or not at all to the pregnancy. These three areas have all been previously associated with all types of stillbirth, including intrapartum fetal death and adverse pregnancy outcome [2, 16–19] and despite ongoing efforts to improve antenatal education, unless there is engagement from the public, as well as acceptance of the risks associated with these lifestyle choices, these efforts will be futile. We suggest the need for a greater public health awareness program with respect to the benefits of healthy eating, exercise, obesity modification and smoking cessation in pregnancy for potential future parents. This information is better imparted pre-conceptually to enable potential parents to optimise their lifestyle pre pregnancy.

Improved antenatal recognition of fetal growth restriction was also identified as an area for improvement. In our study 20% of normally formed infants had a birth-weight less than the 10th percentile for gestational age, and with the exception of one infant this was not recognised antenatally. Fetal growth restriction in utero is associated with perinatal death and consideration should be given to the use of customised growth centiles in order to aid accurate prediction of infants who do not meet their genetic growth potential [16, 20–27]. Identification of risk factors for fetal growth restriction is key and the subsequent management once it is identified may further reduce the risk of intrapartum fetal death [21, 23, 28, 29]. We were unable to identify reasons from the dataset as to why growth restriction was missed so frequently in our cohort. Failure to detect growth restriction in the antenatal period is, however, a finding that is not unique to our study. In the most recent perinatal mortality report from NPEC in Ireland growth restriction was suspected antenatally in just 61% of those infants who were in fact growth restricted [10]. Research from New Zealand and Norway has also identified this as a substantial issue in stillbirth prevention [21, 30].

With respect to the normally formed infants in our cohort, just 60% had a post-mortem examination performed. Most international guidance advocates for the routine use of post-mortem examination and placental histology and it is unclear from the dataset why some infants did not have either test performed [31–33]. One potential reason may have been lack of access to a dedicated perinatal pathologist, but this does not explain why those who were offered a post-mortem declined. There is also clear guidance nationally on when to inform the Coroner of a perinatal death [31]. All unexpected neonatal deaths and intrapartum deaths should be referred through the Coronial system. It is also unclear from the dataset whether the Coroner was informed of some of the cases in this cohort.

Placental histology was available in 91% of cases but as described there was no universal structure to the way placental pathology reports were documented. For some cases, NPEC were given access to the full post-mortem report and this aided with interpretation of findings. There were, however, substantial differences in the way different units reported on placental histology. Some were very detailed while others were not. Formation of a national standardised placental reporting system should be encouraged to aid with accurate diagnosis of cause of death.

One limitation of this study was that data was either missing or unrecorded for some variables. If all data variables were complete, this may have altered the results. This was particularly important for maternal variables that are known risk factors for perinatal death such as smoking or BMI, and in our dataset 10% and 20% of that data was either missing or unrecorded. As the information in our dataset is based on maternal records, it is possible that the risk factor was identified in the antenatal period but not recorded accurately in the maternal record and that may explain some of the missing data.

Despite our results being based on the best available national data on intrapartum deaths and unexpected neonatal deaths, one of the main limitations of this study was the inability to fulfil one of our main aims; to identify if any of these deaths could have been prevented. While we were able to identify risk factors such as the relatively poor antenatal detection of fetal growth restriction, and high rates of maternal obesity and smoking, we were unable to definitively conclude if an improvement through antenatal education of patients and training of healthcare professionals could improve outcomes and prevent deaths.

We were able to use the data provided on the perinatal mortality forms to document the reasons why these infants died, but we did not have access to the mothers’ or infants’ maternity charts, and in particular the labour component. While we were able to document that eight infants died in labour secondary to intrapartum asphyxia and that a further 21 infants died in the neonatal period from HIE it was not possible to conduct a root cause analysis of these cases and this further strengthens the recommendation by NPEC that these cases should all undergo a confidential enquiry process in Ireland [10]. Confidential enquiries are a proven, validated, external review process that have been used extensively in the United Kingdom to investigate maternal death [34] and more recently perinatal fetal and infant death [16]. They are an anonymised, non-judgemental and transparent review process that focus on both good aspects of care as well as identifying areas for improvement. Since 2009, maternity units in the Republic of Ireland through NPEC have been contributing to these maternal death enquiries but as of yet have not contributed to the perinatal death investigations [35]. Development of a confidential enquiry system into intrapartum fetal deaths and unexpected neonatal deaths would provide learning at both local and national levels, and might also help improve the poor public perception of the Irish maternity services. While healthcare professionals have an obligation to provide high-quality evidence based care at all times [36], a confidential enquiry system will identify all areas in the patient journey that need to be improved, including medical and lifestyle factors.

## Conclusions

This analysis reveals valuable information with respect to intrapartum fetal deaths and unexpected neonatal deaths in the Republic of Ireland. Despite using best available national data in this analysis we were unable to ascertain if these deaths could have been prevented. The identification and reduction of preventable intrapartum fetal deaths and neonatal deaths secondary to intrapartum events would head the call from the Each Baby Counts project in the United Kingdom [13]. This project aims to reduce loss of life from intrapartum events by 50% by 2030, and mirrors the World Health Organisation’s Sustainable Development Goal Number 3; to end preventable deaths of new-borns and children under the age of five [37]. We, therefore, highlight the absolute need for a confidential enquiry process in Ireland to ensure that all preventable intrapartum deaths and neonatal deaths are recognised and learned from in a timely manner, but equally to ensure that good aspects of maternity care are reported.

## Abbreviations

BMI: Body Mass Index
CS: Caesarean Section
IPD: Intrapartum Fetal Death
IQR: Interquartile Range
NPEC: National Perinatal Epidemiology Centre
PM: Post-mortem
ROI: Republic of Ireland
SVD: Spontaneous Vaginal Delivery
